# Cavity-Backed Antenna-Coupled Nanothermocouples

**DOI:** 10.1038/s41598-019-46072-4

**Published:** 2019-07-03

**Authors:** Gergo P. Szakmany, Alexei O. Orlov, Gary H. Bernstein, Wolfgang Porod

**Affiliations:** Department of Electrical Engineering, University of Notre Dame, Notre Dame, IN 46556 USA

**Keywords:** Electrical and electronic engineering, Sensors

## Abstract

This paper reports a two-orders-of-magnitude improvement in the sensitivity of antenna-coupled nanothermocouple (ACNTC) infrared detectors. The electrical signal generated by on-chip ACNTCs results from the temperature difference between a resonant antenna locally heated by infrared radiation and the substrate. A cavity etched under the antenna provides two benefits. It eliminates the undesirable cooling of the hot junction by thermally isolating the antenna from the substrate. More importantly, careful cavity design results in constructive interference of the incident radiation reflected back to the antenna, which significantly increases the detector sensitivity. We present the cavity-depth-dependent response of ACNTCs with cavity depths between 1 μm and 22 μm. When constructive interference is maximized, the thermal response increases by 100-fold compared to devices without the cavity.

## Introduction

Long-wave infrared (LWIR) and THz detectors are of special interest due to black-body radiation of objects at room temperature. Applications of these detectors include energy harvesting^1^, target tracking and identification of hidden objects^2^, atmospheric research^3^, biological sensing^4^, and medical diagnostics^4,5^. Antenna-coupled detectors are based on the wave nature of the IR and THz radiation; a nanoantenna receives the freely-propagating electromagnetic waves, and the infrared radiation-induced antenna currents are converted to electrical signals by a bolometer^6,7^, a nanothermocouple (NTC)^8–10^, or a heterostructure backward diode^11,12^.

Antenna-coupled nanothermocouples (ACNTCs) convert the optical energy to electrical signals by Joule heating and the Seebeck effect. The radiation-induced antenna currents increase the temperature of the hot junction of the thermocouple that is located at the center of the antenna, and an open-circuit voltage (V_OC_) is induced, as expressed by

1 $$Voc={\rm{\Delta }}S\cdot {\rm{\Delta }}T,$$

where ${\rm{\Delta }}S$ is the relative Seebeck coefficient of materials that compose the NTC, and $\Delta T$ is the temperature gradient between the cold and hot junctions.

**Figure 1:**
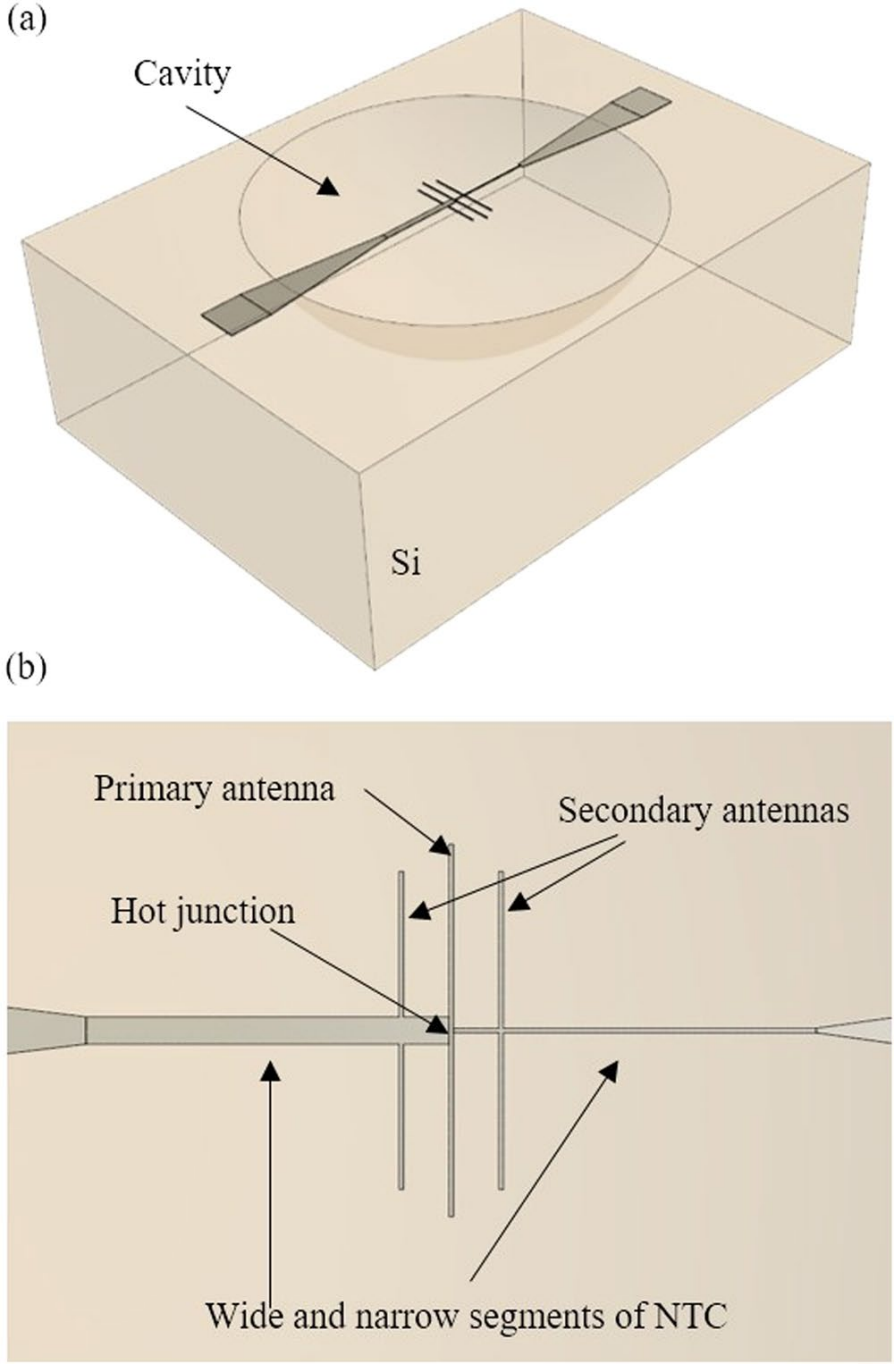
Schematic of the suspended ACNTC. (**a**) Isometric view of the ACNTC and the cavity, (**b**) top view of the primary and secondary antennas and the lead lines of the NTC.

The response of thermal-based antenna-coupled detectors is proportional to the heating of the sensing element (bolometer, NTC) and inversely proportional to the heat removal by the bulk of the chip. Recently, we have demonstrated that thermal insulation of the antenna from the substrate by a SiO_2_ membrane^13^ or suspending over a cavity^14^ increased the thermal response of ACNTCs by a factor of 2.5 times and 20 times, respectively. In our previous work it was observed^14^ that the magnitude of the measured *V*_*OC*_ of the thermally insulated devices is cavity-depth-dependent, and in this work we study the generation of a voltage by suspended single-metal ACNTCs over various cavity depths, as shown in Fig. 1. In particular, we design ACNTCs to detect 28.3 THz electromagnetic waves because the IR source of our experiments is a CO_2_ laser that is operating at this frequency. Therefore, we use half-wave dipole antennas that are fairly narrow band, and their frequency selectivity is tuned by their physical lengths. Because the interference caused by the cavity is also frequency dependent, the geometry of the cavity has to also match the wavelength of the incident radiation. For applications that require broadband detection, e.g., energy harvesting, broadband antennas and matching cavity are required, but is not the focus of this paper.

Experimental and simulation results show that the cavity reflects the incident radiation back to the antennas, and interferences occur between the reflected and incident IR waves resulting in a cavity-depth dependent response.

## Design and Fabrication

### Nanoantenna design

In order to receive the 10.6-μm IR irradiation, the antenna length has to be matched the effective wavelength of the incident electromagnetic waves. Similar to our previous work^8^, COMSOL Multiphysics Simulation Software^15^ was used to determine the resonant antenna length, which was found to be 4.1 μm for our suspended primary dipole antenna. The hot junctions of the NTCs are placed at the center of the antenna where the radiation-induced antenna currents are at maximum^16^. To further increase the heating of the hot junction of the NTC, two nanowires were placed in parallel to the antenna, as shown in Fig. 1. The secondary antennas also resonate with the incident radiation, which contributes additional antenna current and thus heating of the hot junction. As a result, Δ*T* significantly increases, and a larger Seebeck voltage is induced. We have found that maximum temperature increase is achieved when the secondary antennas are 500 nm away from the primary antenna, and their lengths are 3.5 μm. This paper reports mostly on the effects of the cavity parameters whereas details of the effects of the primary and secondary antennas are reported elsewhere^17^.

### Nanothermocouple design

The heat produced by the nanoantenna is converted to electrical signals by the NTC. Usually, thermocouples are constructed from two different conductors with different absolute Seebeck coefficients (ASC). However, we have recently demonstrated^18^ that nanowire thermocouples can be constructed from a single metal with a cross-sectional discontinuity at the hot junction. Single-metal NTCs are based on the size-dependent ASC of metals at nanoscales. When the physical sizes (width and thickness) of metallic conductors are comparable to the electron mean free path, all transport properties reduce compared to their bulk values^19–22^ due to the increase of grain boundary^23^ and surface scattering^24,25^ of electrons. Size quantization effects^26^ are negligible in metals at these dimensions due to the very short Fermi wavelength^27^. As a result, the ASC of metal nanowires are reduced compared to their bulk values^21,28–32^. We have previously shown^21^ that when narrow and wide nanowire segments are joined at one end from the same metal, the reduction of the ASC of the narrow wire segment is more prominent than in the wider wire segment. The resulting size-dependent difference in the ASC leads to a measurable difference in the relative Seebeck coefficient (RSC)^18^, thus creating a thermocouple junction at the size discontinuity. In this work, we used single-metal NTCs to reduce the fabrication complexity of the devices. We built single-metal NTCs from 65 nm and 330 nm wide Pd wire segments. Because the geometry of the NTC in ref.^18^ is the same as in this work without the cavity, the expected RSC of our single-metal NTC is about 1.2 μV/K.

### Fabrication

The suspended ACNTCs were fabricated on a high-resistivity Si wafer (>20,000 Ω-cm) that provides sufficient electrical isolation between the devices without the need for an oxide layer. The 200-nm-thick Au bonding pads and lead lines for electrical and IR measurements were fabricated by using optical lithography.

A Raith 5200 EBPG electron beam lithography (EBL) system was used to pattern the ACNTCs. The receiving elements of the ACNTC were patterned into a methyl methacrylate (MMA) and polymethyl methacrylate (PMMA) resist structure. The MMA layer was pre-exposed with a deep UV light source, providing a necessary undercut in the resist profile to aid in the lift-off process. Development of the exposed patterns were performed in a mixture of isopropanol (IPA) and methyl isobutyl ketone (MIBK) with a 3:1 ratio with 1.5% methyl ethyl ketone (MEK)^33^ for 45 s. The 45-nm-thick Pd layer that forms the ACNTCs was electron beam evaporated, and 1-methyl-2-pyrrolidinone (NMP) was used for lift-off.

Next, the cavities were formed under the finished ACNTCs. A 6 μm x 6 μm square, centering the hot junction of the ACNTCs, was exposed with EBL into a MMA/PMMA resist layer to form an etch window. After development, the native oxide layer on the Si wafer was removed by reactive ion etching using a mixture of CHF_3_ and O_2_ for 30 s at 300 W. Then, the cavities were formed by using gaseous XeF_2_ to etch the Si substrate under the ACNTCs. The physical geometry (depth and width) of the cavity is controlled by the pressure and etch duration. Suspended ACNTCs with cavity depths ranging between 1 μm and 22 μm were fabricated.

**Figure 2:**
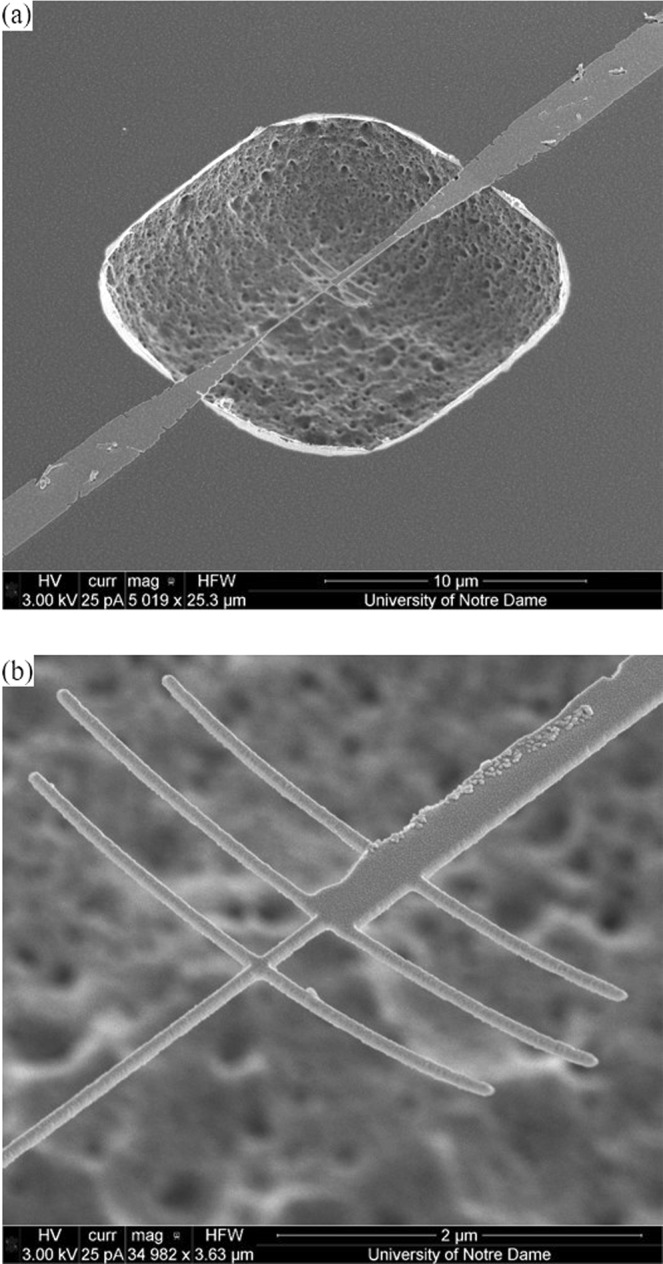
SEM images of the suspended ACNTC. (**a**) The antenna structure and the cavity. (**b**) Enlarged view of the primary and secondary antennas and the hot junction. The antennas and the narrow section of the NTC are 65 nm wide, and the wider segment of the NTC is 330 nm wide.

A finished ACNTC with thermal insulation is shown in Fig. 2. The ACNTCs are nominally identical for all cavity depths. The primary antenna is 4.1 μm long, and the secondary antennas are 3.5 μm long. The antennas and narrow segment of the NTC are all 65 nm wide, while the width of the wide segment of the NTC is 330 nm.

## Results

In this section, we discuss the characterization of the finished devices. The V_OC_ response to IR excitation of the ACNTC was recorded for various cavities. The cavity depths from each fabrication set were measured to account for the lack of precision in the XeF_2_ etch step.

### Cavity depth

**Figure 3:**
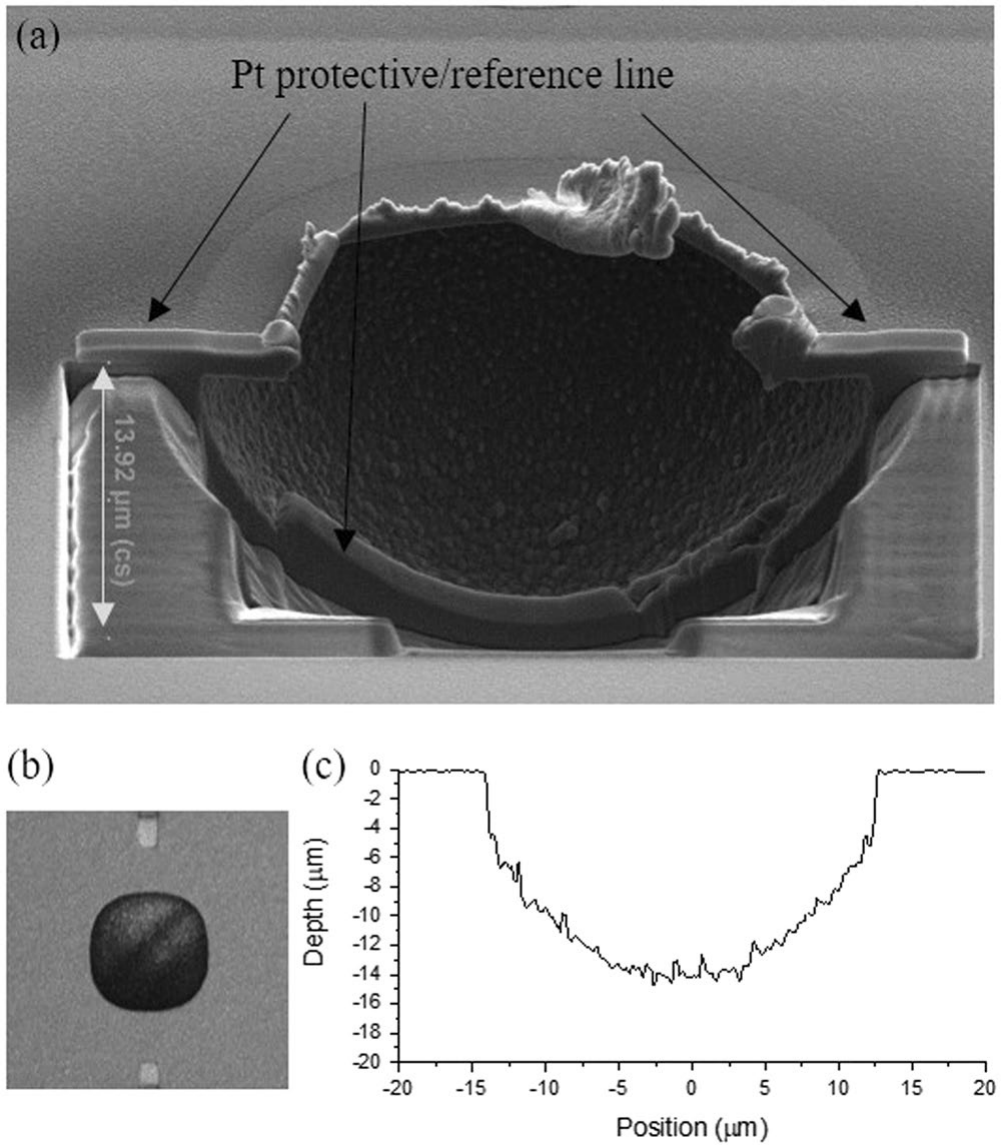
Cavity profiles imaged by (**a**) FEI Helios FIB, and (**b**) Olympus LEXT 4100. (**c**) Cavity profile by the confocal microscope.

There are several ways to determine the cavity depth. Focused-ion beam (FIB) provides a detailed view of the cross section of the cavity, including depth, width, and roughness, but provides a cross section along only one line and is destructive. Hence, this method can be used only after the devices were tested for the IR response. As an alternative, we used the Olympus LEXT OLS4100 laser confocal microscope to determine the cavity depth and its profile, and used the FIB to confirm the results on selected samples. Figure 3a shows the cross section of a cavity created with a FIB. Figure 3b,c show the image and profile of the same cavity acquired by the Olympus LEXT, and by comparing Fig. 3c to Fig. 3a the obtained cavity depths are the nearly identical.

Figure 3a also shows that the side of the cavity has to be milled to reveal the cavity profile when using the FIB, and as a result, the sample is damaged, while the confocal microscope can perform measurements without damage.

Note, while the Olympus LEXT is capable of determining the cavity depth when ACNTCs are present above the cavity, the laser scan is obstructed by the nanoantenna and NTC lead lines at the center of the cavity and create a noisy cavity profile. Therefore, a bonding pad pair without any devices was dedicated for cavity profile measurements.

In order to characterize the uniformity of the XeF_2_ etch, cavities were fabricated on a chip without any ACNTCs by the fabrication process presented above. The mean depth and width were 9.75 μm and 25.39 μm, respectively. The dimensions of the cavities varied less than 0.65% over the entire chip within one fabrication run; see Supplementary Information for details.

### IR response

The fabricated devices were wire-bonded to a chip carrier and inserted into a chip socket on a PCB with BNC connectors for electrical testing under IR irradiation. The PCB was attached to a translation stage having three degrees of freedom. The LWIR source is a linearly polarized CO_2_ laser operating at 28.3 THz. The V_OC_ response of an ACNTC is at maximum when the polarization of the incident laser beam is parallel to the axis of the antenna as expected from antenna theory^16^. We used a half-wave plate to set the co-polarization state by rotating the polarization of the laser beam. *V*_*OC*_ was measured using a high-input-impedance differential amplifier and a lock-in amplifier synchronized with the laser beam chopper.

**Figure 4:**
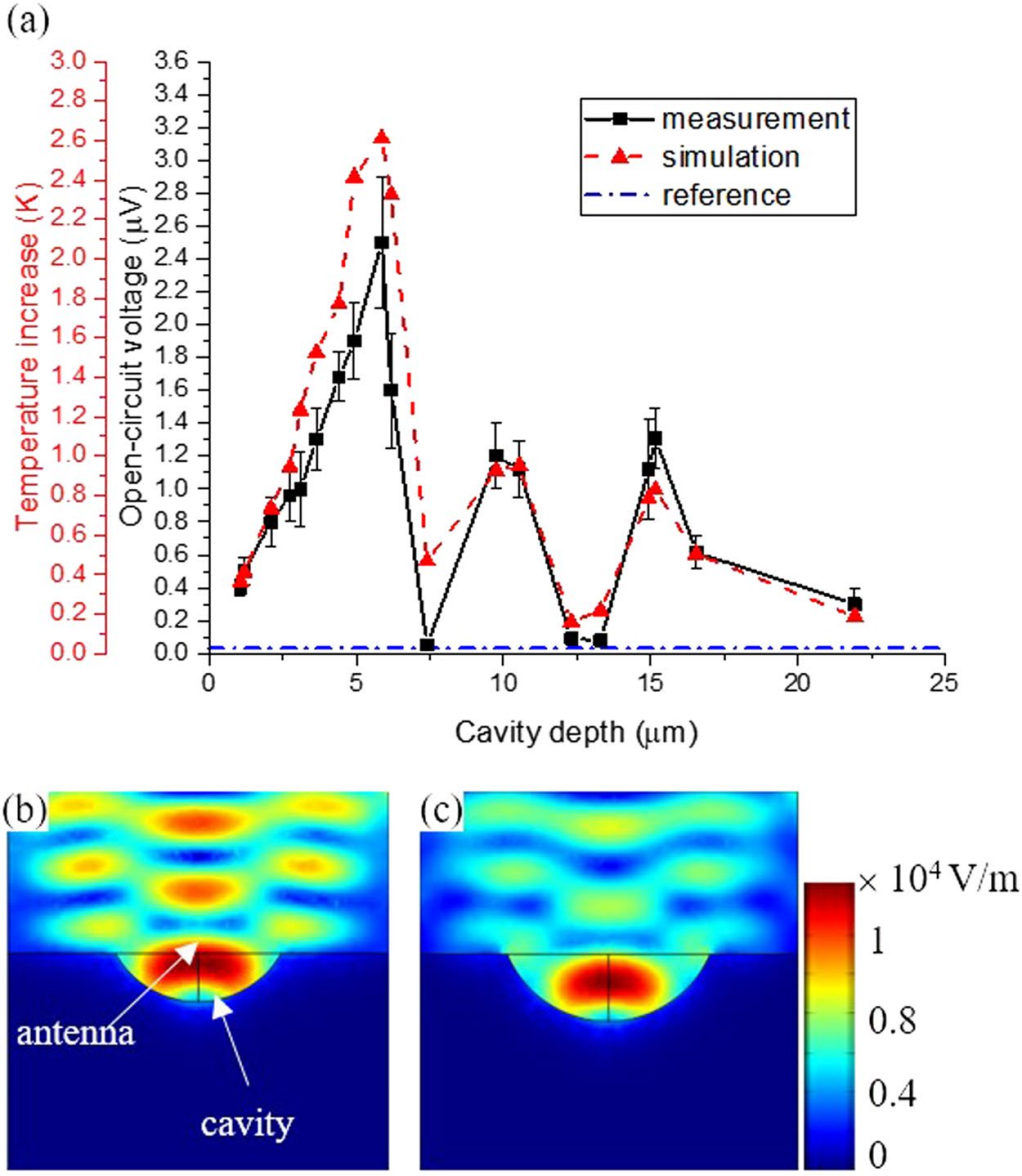
(**a**) Measured and simulated responses of the suspended ACNTCs as a function of cavity depth. The measured V_OC_ and the simulated temperature increases are proportional to the relative Seebeck coefficient of the NTC that is 1.2 μV/K. Simulated electric field intensity across the center of the cavity (**b**) for the first peak, and (**c**) for the first valley of (**a**). In (**b**), the antenna is immersed in a field maximum, whereas in (**c**) the antenna is immersed in a field minimum.

Figure 4 shows the measured *V*_*OC*_ as a function of cavity depth. Each data point represents the average value of the responses of at a minimum 10 nominally identical devices, and the error bars are the standard deviation. Figure 4 shows that V_OC_ oscillates with cavity depth, and the distances between the adjacent maxima or minima are about 5 μm, which is about a half-wavelength of the incident radiation.

This behavior can be explained by the shape of the cavities. The isotropic nature of the Si etch in XeF_2_ results in a spherical cavity, as shown in Fig. 3. Therefore, the cavity does not just simply thermally insulate the devices from the substrate by removing the Si and replacing it with air, which has a smaller thermal conductivity,$\,{\kappa }_{air}=0.025\,W{m}^{-1}\,{K}^{-1}$, than Si, ${\kappa }_{Si}=134\,W{m}^{-1}\,{K}^{-1}$. The wall of the cavity also acts as a mirror and reflects the incident radiation. The reflected and incident radiations at the antenna interfere, and the responses of the ACNTCs oscillate with cavity depth.

Figure 4 also shows that the response of the ACNTCs when the cavity depth is 5.6 μm deep is about 100 times larger than that of ACNTCs without any thermal insulation on a substrate, as reported previously^8,34^. That response is shown by the dash-dot line at a constant value of 30 nV.

## Discussion

In order to support the experimental results, the expected temperature increase by the antenna was calculated using COMSOL Multiphysics Simulation Software^15^ for various cavity depths. The simulations take into account the induction of antenna currents due to the incident IR radiation and resulting heating. It also includes the heat flow along the antenna and lead lines of the NTC, and into the surroundings of the antenna. The simulated temperature increases as a function of cavity depth are shown in Fig. 4.

The double left axis in Fig. 4 indicates the link between the measured *V*_*OC*_ and the simulated temperature increase. As Eq. 1 shows, the two quantities are proportional by the relative Seebeck coefficient. We used ${\rm{\Delta }}S=1.2\,\mu V/K$, because the widths of the narrow and wide segments of the NTCs are the same as in ref.^18^. Therefore, the scales on the *V*_*OC*_ and temperature increase axis represent this proportion. Figure 4(b,c) show the electric field intensity across the center of the cavity for the first interference peak and valley from Fig. 4(a). When simulations and measurements show maximum ACNTC response, constructive interference occurs and the intensity of the electric field is large at the antenna position. When the simulations and measurement show minimum ACNTC response, the intensity of the electric field is minimal due to destructive interference. Figure 4 shows that the experimental and simulation results are in excellent agreement, and confirm the oscillating antenna behavior as a function of cavity depth.

Note that our initial model of the cavity was spherical for all cavity depths, which is a good approximation for cavities deeper than 7 μm, as shown in Fig. 3. However, the bottom of shallower cavities is flat, and the simulation overestimated the temperature increase because a spherical surface directs more IR radiation to the antenna than does the flat cavity bottom. Therefore, the physical dimensions of the cavities, widths, depths, and profile were extracted from Fig. 3c. Then the 3D model of the cavity was created with the Autodesk Fusion 360 CAD program, and imported to COMSOL where the remaining part of the structure was modelled and simulated. The temperature values on Fig. 4 represent this modeling.

The small deviation between simulation and mean values found in the experiment demonstrate that the ACNTC responses are highly dependent on cavity depth. Conversely, the large standard deviation of nominally identical devices can be explained by fabrication errors including the roughness of the cavity surface after XeF_2_ etching. Loss of the primary and secondary antenna planarity due to residual stress causes further loss in detection efficiency resulting in a smaller *V*_*OC*_ than that predicted by the simulations.

## Summary and Conclusion

We show that thermal insulation of ACNTCs from the substrate by suspending them over a cavity increases the device response by almost 100-fold. The optimized shape and geometrical dimensions of the cavity are essential to reach such an increase. The cavity does not just eliminate most thermal losses to the Si (leaving only thermal conductance through the lead lines and air), but it also reflects the electromagnetic waves and causes interferences at the antenna. For example, when the depth of the cavity is 7.5 μm, the *V*_*OC*_ response of such devices is only 1.5 times larger than devices without any thermal insulation on a substrate, while with cavity depth of 5.6 μm, the increased response is 100 times larger.

This technique might be also useful for other antenna-coupled detectors, even for those that are not based on thermal effects, because the radiation-induced antenna current increases with properly designed cavities due to constructive interference. As a result, the rectified signal and hence the device performance can be increased. In this work, the experimental and simulation results represent the response of ACNTCs operating at 28.3 THz. Because the oscillating cavity behavior is proportional to the wavelength of the incident radiation, the cavity size has to be adjusted for different wavelengths.

Note that the structure of our antenna configuration is very similar to a spherical reflector IR antenna^35,36^; however, the distance between the feed antenna (primary and secondary antennas) and the spherical reflector (cavity wall) is less than 2λ. Thus, the reflector is in the near-field of the feed antenna, and as a result our sensor size is about five times smaller.

## Supplementary information

Cavity-Backed Antenna-Coupled Nanothermocouples

## Data Availability

The datasets generated during and/or analyzed during the current study are available from the corresponding author on reasonable request.
