# Supplementary material for: Cavity-Backed Antenna-Coupled Nanothermocouples
Source: Sci Rep. 2019 Jul 3;9:9606. doi: 10.1038/s41598-019-46072-4 (PMC6610083; doi:10.1038/s41598-019-46072-4)
Supplement: Supplementary file 1 — Cavity-Backed Antenna-Coupled Nanothermocouples [file 41598_2019_46072_MOESM1_ESM.docx]

Supplementary Information

Cavity-Backed Antenna-Coupled Nanothermocouples

Gergo P. Szakmany, Alexei O. Orlov, Gary H. Bernstein, Wolfgang Porod

In order to characterize the uniformity of the XeF_2_ etch, 13 cavities without ACNTCs were fabricated as described in the Fabrication Section of the main text. The cavities are uniformly distributed in a 3.5 mm by 0.5 mm area. Cavities were etched for either 40 s or 80 s. After removing the photoresist, each cavity depth was measured by an Olympus LEXT 4100 laser confocal microscope. The depth of each cavity from the two chips is shown in Table S1. The mean depth of the 40-s-long etch is 9.75 μm and the mean depth of the 80-s-long etch is 25.39 μm. The standard deviations from the mean are 0.65% and 0.66%.

Table S1. Measured cavity depths used to characterize XeF_2_ etch uniformity within a chip.

| Cavity # | Depth (μm)  40 s etch | Depth (μm) 80 s etch |
| --- | --- | --- |
| 1 | 9.741 | 25.392 |
| 2 | 9.741 | 25.385 |
| 3 | 9.748 | 25.378 |
| 4 | 9.758 | 25.402 |
| 5 | 9.743 | 25.386 |
| 6 | 9.751 | 25.391 |
| 7 | 9.747 | 25.394 |
| 8 | 9.756 | 25.391 |
| 9 | 9.741 | 25.384 |
| 10 | 9.749 | 25.387 |
| 11 | 9.752 | 25.389 |
| 12 | 9.756 | 25.400 |
| 13 | 9.759 | 25.384 |
| Mean | 9.75 | 25.39 |
| Standard deviation | 0.65% | 0.66% |
